# Sub-optimal temperatures lead to altered expression of stress-related genes and increased ‘C*andidatus* Liberibacter solanacearum’ accumulation in potato psyllid

**DOI:** 10.3389/finsc.2023.1279365

**Published:** 2024-01-12

**Authors:** Tonja W. Fisher, Joseph E. Munyaneza, Judith K. Brown

**Affiliations:** ^1^ School of Plant Sciences, The University of Arizona, Tucson, AZ, United States; ^2^ United States Department of Agriculture-Agricultural Research Service (USDA-ARS), Yakima Agricultural Research Laboratory (YARL), Wapato, WA, United States; ^3^ School of Plant Sciences University of Arizona, Tucson, AZ, United States

**Keywords:** *Bactericera cockerelli*, ‘*Candidatus* Liberibacter solanacearum’, RNAseq, thermal stress, transcriptome

## Abstract

**Introduction:**

The potato psyllid *Bactericera cockerelli* is the insect vector of the fastidious bacterium ‘*Candidatus* Liberibacter solanacearum’. The bacterium infects both *B. cockerelli* and plant species, causing zebra chip (ZC) disease of potato and vein-greening disease of tomato. Temperatures are known to influence the initiation and progression of disease symptom in the host plant, and seasonal transitions from moderate to high temperatures trigger psyllid dispersal migration to facilitate survival.

**Methods:**

‘*Ca*. L. solanacearum’ -infected and uninfected psyllids were reared at previously established ‘permissible’, optimal, and ‘non-permissible’ and temperatures of 18°C, 24°C, and 30°C, respectively. Gene expression profiles for ‘Ca. L. solanacearum’-infected and -uninfected adult psyllids reared at different temperatures were characterized by Illumina RNA-Seq analysis. Bacterial genome copy number was quantified by real-time quantitative-PCR (qPCR) amplification.

**Results:**

Relative gene expression profiles varied in psyllids reared at the three experimental temperatures. Psyllids reared at 18°C and 30°C exhibited greater fold-change increased expression of stress- and ‘*Ca*. L. solanacearum’ invasion-related proteins. Quantification by qPCR of bacterial genome copy number revealed that ‘*Ca*. L. solanacearum’ accumulation was significantly lower in psyllids reared at 18°C and 30°C, compared to 24°C.

**Discussion:**

Temperature is a key factor in the life history of potato psyllid and multiplication/accumulation of ‘*Ca*. L. solanacearum’ in both the plant and psyllid host, influences the expression of genes associated with thermal stress tolerance, among others, and may have been instrumental in driving the co-evolution of the pathosystem.

## 1 Introduction

Zebra chip (ZC) is a recent and economically important disease of potato, occurring in Mexico, Central America, United States of America (U.S.), and New Zealand (1–5). The potato/tomato psyllid, *Bactericera cockerelli* (Sulc.) (so. Hemiptera: order Homoptera), is native to North America (6, 7) and occurs in Central America, the western U.S., and as far north as Canada (5). Infestations by *B. cockerelli* have long been associated with psyllid yellows disease of potato and tomato, an uncharacterized disorder characterized by foliar chlorosis, stunting, and yield loss (8–10). Most recently, the emergent obligate, plant pathogenic bacterium, ‘*Candidatus* Liberibacter solanacearum’ belonging to the Alphaproteobacteria (11), also known as ‘*Ca*. Liberibacter psyllaurous’ (12), has been identified as the agent associated with zebra chip disease of potato and vein-greening (13) diseases of potato and tomato crops, respectively. The bacterium infects other solanaceous crops, including eggplant, pepper, and tobacco, and weed species (3, 5, 11, 14–20) as well as non-solanaceous crops (21–23).

Insect metabolic rate is known to be highly sensitive to temperature, and the concomitant acceleration of metabolism associated with increased temperature exposure can result in higher nutrient consumption, lipogenesis, and increased growth and development rates (24–27) that in turn can lead to increased population size and downstream wide-spread disease outbreaks. Indeed, specific temperature requirements have been reported for seasonal establishment of the psyllid vector and outbreaks of Liberibacter-associated diseases of citrus, and potato and tomato (5, 28–31). Also, reduced acquisition/transmission efficiency of ‘*Ca*. L. asiaticus’ (CLas) in *Diaphorina citri* nymphs and adults was reported to occur under sub-optimal temperatures (32). Further, reduced transmission rates have been demonstrated for bacterial pathogens of humans and other mammals, including *Yersinia pestis*, by the Oriental rat flea *Xenopsylla cheopis* (33), at non-optimal temperatures (34). In contrast, in human pathosystems, increased pathogenicity and disease severity of *Legionella pneumophila* and *Shigella* sp. have been correlated with periods of elevated temperatures (35–37).

Functional “omics” characterization of insect physiological responses and tolerance to environmental stress physiology is facilitated by comparative analyses of the proteome, transcriptome, metabolome, considered the primary or core pillars of response hierarchies (38). Several hemipteran phloem-feeding insects, including psyllids (39, 40) and whiteflies (41), have a specialized alimentary structure, or filter chamber that consists of a substrate permeable membrane channel on the hydrophobic interior and size-exclusion filter on the extracellular side of the membrane that allows for rapid water transport. This structure functions in osmoregulation essential for insects with high-volume liquid diets, cryoprotection, and anhydrobiosis, the latter, required to survive thermal and other environmental stresses (42, 43). In the whitefly, *Bemisia tabaci* (Genn.) and potato psyllid, movement of water through opposing alimentary tract tissues within the filter chamber involves the integral transmembrane protein, aquaporin (44, 45). In whiteflies, it has been shown to be expressed in the filter chamber and hindgut of early instar nymphs and adults where it relieves osmotic stress by stabilizing water balance through recycling and excretion functions (45). Down-regulation of aquaporin homologs encoded by aphids (46), *B. tabaci* (45, 47), and psyllids (48) resulted in disruption of normal osmoregulation functions, and for latter two insects, increased nymphal mortality and/or reduced lifespan of adults. In *D. citri*, knockdown evidence of differential changes in the metabolome and increased uric acid accumulation (48), which increases free radical-scavenging activity in hemolymph (49).

Osmotic stress has been shown to be involved in regulating expression of the Ras-like GTPase Rap 1 (guanosine triphosphatase), which functions as conserved molecular switch involved in downstream regulation of effector molecules that regulate cell growth and differentiation, secretion, cell adhesion and morphogenesis. In most organisms, Rap1 associates with effectors that regulate signaling pathways governing actin cytoskeleton and adhesion molecules (50) that modulate and control critical cellular processes. Further, actin cytoskeleton dynamics is known to be altered by certain pathogens to gain cellular entry, which triggers Rap 1 and other molecules that function in pathogenesis responsive processes and in dsRNA uptake (51, 52).

Other strategies for combatting biotic stress reside within the insect innate immune system Toll and immune deficiency (IMD) pathways that are involved in the genesis of antimicrobial peptides. They regulate the host response to Gram-negative bacterial infection by recognizing diaminopimelic acid (DAP)-type peptidoglycans that in turn interact with peptidoglycan recognition proteins LC (PGRP-LC) and LE receptors. Among the best-studied downstream components of the IMD pathway are *Drosophila* homologs of TAK1, IKK, and JNK kinases (53). Finally, lysosomes, a type of membrane bound organelle, contain hydrolytic enzymes that digest or degrade endogenous and exogenous sub-stances, and importantly, are responsible for nutrient sensing and storage, and retrieval. In these latter functions they participate in fusion and fission events with other organelles and the plasma membrane, including pino- and micropinocytotic vesicles and phagocytic vacuoles by which exogenous substances are transported into the cell. Lysosomes also participate in core physiological and pathogen-related processes in eukaryotes, including animal species, and invertebrates such as insects [see references in 54]. Cathepsins are proteases involved in the innate immune response in insects, for example, in the ACP midgut Cath-L (but not Cath-O) has been associated with the expression of Toll pathway constituents in CLas-infected psyllids, presumably to combat CLas invasion (55).

Despite recent advancements, studies to elucidate the multi-trophic interactions of biotic and abiotic stress enhancers (i.e., temperature, pathogen infection, other) in insect vector-pathosystems at gene expression and other functional “omics” levels, are limited (56). Yet, these studies are important and are evidenced by a study that suggests the negative effects of ‘*Ca.* L. solanacearum’ on potato psyllid fecundity were most likely due to the direct effects of the bacteria on the insect host and not host-plant associated (57). Further, understanding the dynamics of biotic-abiotic stress interactors could lead to new biopesticidal technologies to complement IPM functional genomic insights into psyllid-Liberibacter pathosystem, since biochemical, cellular, and functional genomic processes that could help guide the exploitation of RNA interference (RNAi) technologies have been limited. The paucity of relevant information has continued to hinder the identification of targets, that if knocked down, could inhibit key steps of ‘*Ca*. Liberibacter’ pathogenesis and circulative or systemic infection of the psyllid vector, while also reducing the number of bacteriliferous offspring.

‘*Ca*. Liberibacter’-associated diseases are managed primarily by chemical pesticide applications aimed at reducing the vector population size to concomitantly lower the rate of pathogen transmission to plant hosts, a number of complementary approaches have been implemented or are under consideration. These include breeding for host plant resistance (5), augmentative biological control (58), RNA interference (RNAi) technology for knockdown gene expression (59-61), and CRISPR-Cas editing approaches (62, 63).

The goal of this study was to investigate the prospective effects of temperature on potato psyllid vector fitness in different climatic and ecological scenarios using functional genomic analysis of gene expression together with quantitative PCR detection of ‘*Ca*. L. solanacearum’ to monitor pathogen load in adult psyllids post-exposure to temperatures representing ambient, extreme heat, or cold conditions. The rationale is that knowledge derived from gene profiling, in relation to ‘*Ca*. L. solanacearum’ accumulation in potato psyllids post-temperature treatments, could guide disease control efforts that focus on disrupting ‘*Ca*. L. solanacearum’ gut invasion and multiplication therein, and/or downstream systemic infection processes in the circulative transmission pathway to reduce bacterial transmission by the potato psyllid vector.

## 2 Materials and methods

### 2.1 Psyllid colonies

Certified pathogen-free potato mini-tubers (derived from tissue culture) of variety Atlantic were obtained from CSS Farms Inc. (Colorado City, CO, USA). Potato psyllid colonies were initially established from individuals collected from potato fields in Texas and Washington, as previously reported (40). Individuals from each colony were tested for ‘*Ca*. L. solanacearum’ presence by PCR amplification of the 16S rRNA gene, using an established method (11). Psyllids and ‘*Ca*. L. solanacearum’ were previously identified as the “central” and ‘*Ca.* L. solanacearum B’ haplotypes, respectively (5, 64). Colonies of ‘*Ca*. L. solanacearum’-infected and -uninfected potato psyllids were established and maintained in a separate growth chamber at the USDA-ARS facility in Wapato, WA (USA).

### 2.2 Temperature treatments

Temperature studies were conducted in a growth chamber (Percival Inc., Boone, IA) maintained at the following three temperatures, 18°C, 24°C, and 30°C. All chambers were maintained at 70% RH and light was provided at a 12:12 (L: D) h photoperiod. Plants used in this study were housed in small, individual hoop cage as described previously (5). Briefly, metallic wires were inserted into the four corners of the pot to create a frame over the plant and the frame was covered with insect screen fabric secured to the base of the pot with a large rubber band. Each experimental replicate consisted of 10 plants per temperature. Ten female and ten male adult psyllids (infected or uninfected) were placed on each plant and placed in a growth chamber. Adult psyllids were allowed to mate and reproduce. Approximately 30 days after psyllid addition, the progeny adult psyllids (n=100) were collected, crushed in Trizol, and stored at -80°C until shipping on dry ice for RNAseq library preparation at the Institute of Biological Chemistry, Washington State University, Pullman, WA, USA.

### 2.3 Quantitative polymerase chain reaction amplification

DNA was extracted and purified from ‘*Ca*. L. solanacearum’-infected *B. cockerelli* adults transferred to potato plants maintained in a growth chamber, at 18°C, 24°C, or 30°C, respectively. Quantitative-PCR (qPCR) was carried out using Taqman (hydrolysis) probes and primers designed to detect the ‘*Ca*. L. solanacearum’ outer membrane protein gene (*omp*; GenBank accession FJ914617) and *B. cockerelli* cytochrome oxidase 1 (*COX;* GenBank accession EF372597) reference gene (PrimeTime qPCR Assay 6-FAM/ZEN/IBFQ, synthesized by Integrated DNA Technology, IDT, Iowa, USA) (65). The experiment consisted of two biological replicates and three technical replicates with two negative controls (water instead of template; uninfected *B. cockerelli* total DNA) and one positive experimental control that consisted of a fragment of the ‘*Ca*. L. solanacearum’ 16S rRNA target and the *B. cockerelli* reference gene, respectively, cloned into the pGemT-Easy cloning vector (Promega) (65). A ten-fold serial dilution (to 10-^6^) of each linearized plasmid containing the respective verified, cloned insert was used to establish the standard curve. Each qPCR reaction contained 10 μl PCR Master Mix (Applied Biosystems) and 15 ng total DNA in 20 μl, adjusted with DNase/RNase-free water for *omp* and *COX.* Reactions contained 500 nM of each primer, 250 nM of probe (in 1 μl 20X Prime Time Assay, IDT). The qPCR amplification was carried out using a StepOnePlus™ Real-Time PCR System (Applied Biosystems, CA). Cycling conditions consisted of one cycle at 50°C for 2 minutes, followed by one cycle of 95°C for 10 minutes, then 40 cycles of 95°C for 15 seconds and 60°C for 60 seconds. To verify the presence of qPCR products in reactions, amplicons were visualized on a Gelred (Biotium)-stained 1% agarose gel in Tris-Acetate-EDTA buffer, pH 8.0, post-amplification.

### 2.4 Total RNA extraction, library construction and Illumina sequencing of adult psyllids

Total RNA was purified from ‘*Ca*. L. solanacearum’-infected and uninfected *B. cockerelli* adults grown at different temperatures as shown, 8 replicates from each treatment, for a total of 48 samples. For RNA extraction, 0.3 ml chloroform were added to 1 ml Trizol homogenate, followed by vigorous shaking for 30s and incubation for 3min at room temperature. Tubes containing samples were centrifuged at 12,000 ×g for 15 min at 4°C to separate organic and aqueous phases. The aqueous phase (200-250μl) was transferred to a sterile, RNase-free tube. An equal volume of 100% EtOH was added to each, and the contents were gently mixed. Samples were purified using the RNeasy Mini Kit (Qiagen, Valencia, CA), according to the manufacturer’s protocol. The quality and quantity of RNA was assessed using a NanoDrop 2000 Spectrophotometer (Thermo Scientific, Wilmington, DE). The ratio of absorbance at 260 nm and 280 nm was used to assess the purity of DNA, and ratios >2.0 were considered optimal. The RNA quality was analyzed on an Agilent 2100 Bioanalyzer (Agilent Technologies Inc., Santa Clara, CA). Samples with an RNA Integrity Number (RIN) value greater than or equal to 8 were selected for preparing the sequencing libraries (Agilent Technologies Inc., Santa Clara, CA). The poly-A RNA was isolated from 2 μg of the total RNA using Dynal oligo (dT) beads (Invitrogen) according to the manufacturer’s protocol. Following purification, the mRNA was fragmented by zinc treatment at 94°C for 5 min, and reverse-transcribed for first-strand cDNA synthesis using SuperScript II reverse transcriptase (Invitrogen). Random primers were added, then second-strand cDNA synthesis was carried out. The ends were repaired, phosphorylated, and an adenine base was added to the 3’-end of the blunt phosphorylated DNA fragments. Illumina adapters with unique indexes were ligated to the fragments, according to the Illumina’s TruSeq RNA Sample Preparation Guide (Illumina). The products were purified for the section of approximately 250 bp in size using Qiaquick Gel Extraction Kit (Qiagen). The cDNA fragments were amplified following the addition of PCR Master Mix and PCR Primer Cocktail (Illumina) to ligate the adapters, for 30 s at 98°C followed by 15 cycles of 10 s at 98°C, 30 s at 60°C, 30 s at 72°C and a final elongation step of 5 min at 72°C. The products were purified using the QIAquick PCR Purification Kit (Qiagen) to create an Illumina paired-end library. Library quality was assessed using a Bioanalyzer DNA 1000 Chip Series II (Agilent).

The libraries were quantified by qPCR amplification, prior to clustering using the protocol recommended by the manufacturer (Illumina). The library was diluted to a final concentration of 10 nM in elution buffer (Qiagen). Samples were diluted to 2nM with 1M NaOH and held at 25°C for 2 min before transferring 2.5 μl into 497.5 μl of HT1 (high salt buffer supplied with cluster kit) to yield a final concentration of 10 pM. One hundred and twenty microliters were transferred to a 200 μl strip tube and applied for cluster generation in a flow cell on a cBOT (Illumina). The flow cell was loaded onto the Illumina Hiseq 2000 instrument, according to the manufacturer’s instructions. Sequencing was carried out at Macrogen with 50bp single read format according to the manufacturer’s instructions.

### 2.5 Bioinformatics analyses

The Illumina raw reads obtained for the ‘*Ca*. L. solanacearum’-infected and -uninfected adult and nymph whole body potato psyllids subjected to the three temperature treatments (64) were used to reassemble a transcriptome, to maximize quality and coverage of the *de novo* assembled transcript data. Briefly, three different assemblers were implemented, using the respective default settings for each: CLC Genomics Workbench v6.5.1 (http://www.clcbio.com), SOAP *de novo* trans v1.03 (66), and EBARDenovo v2.01 (67). The EvidentialGene algorithm was used to generate consensus sequences from the individual assemblies (68). A consensus was determined and compiled for each assembly. The resulting contigs and read counts per library were analyzed using Transcriptome Computational Workbench v 1.6.8 (TCW) software (69). The resultant 25,634 potato psyllid Illumina transcript database was consulted as the reference transcriptome for mapping reads.

Raw data was normalized, and subsequent data was processed using the ArrayStar suite of programs (DNASTAR, Madison, WI). Raw data was normalized using reads per kilobase per million mapped reads (RPKM) method. The processed reads were mapped with the Q-Seq in ArrayStar v14.0 (DNASTAR, Madison, WI) using the default settings. The analysis consisted of 8 replicates. The TCW ‘Filters Query’ was used to query the KEGG (Kyoto Encyclopedia of Genes and Genomes) assignments by exporting the translated open reading frames of selected contigs. Contigs were mapped to the respective biochemical pathways available through the KEGG Automatic Annotation Server (KASS). The bi-directional best hit (BBH) method that implements BLAST to query a set of orthologous groups in KEGG GENES, was used to identify the respective orthologs in KEGG Orthology (KO) and the Enzyme Commission (EC) distributions were determined using the online tool, available at http://www.genome.jp/kegg/kaas/ (50). Recently annotated *Diaphorina citri* sequences (70) were used to supplement potato psyllid transcript identifications using the database available in the Brown lab [database in ref 64].

## 3 Results

### 3.1 Altered ‘*Ca*. L. solanacearum’ accumulation in response to temperature

**Figure 1:**
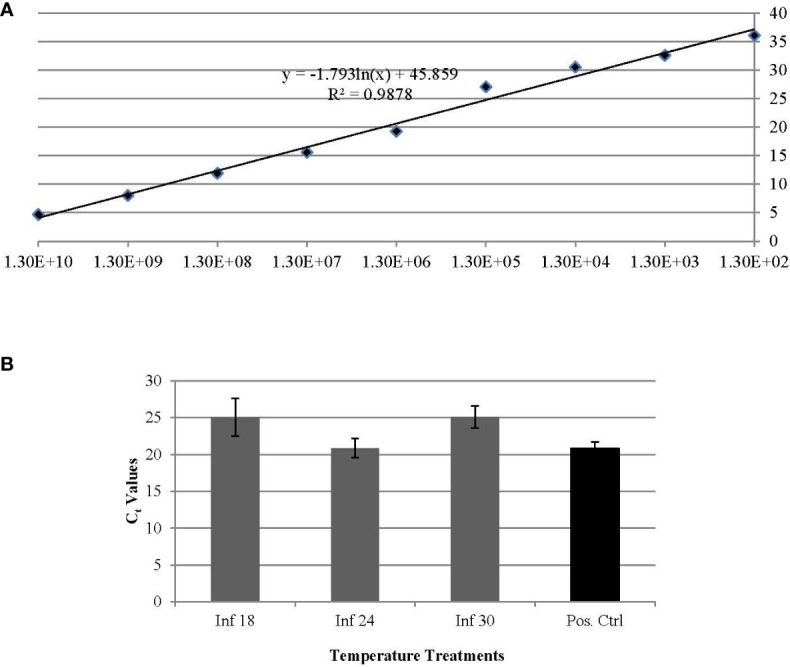
**(A, B)**. Standard curve based on relationship of Ct (y-axis) and copy number (x-axis) per 25 ng of DNA established for quantification of ‘*Ca.* L. solanacearum’ in potato psyllids **(A)**. The average Ct values of experimental potato psyllids reared at 18°C, 24°C, and 30°C and the average Ct value of potato psyllids maintained in laboratory colonies **(B)**. Potato psyllids reared at the two temperature extremes, 18 and 30°C, had one order of magnitude lower number of copies of ‘*Ca*. L. solanacearum’ DNA, compared to those reared at the optimal temperature of 24°C.

Results from qPCR analysis indicated that long term temperature exposures, e.g. ~ 30 days, comparably influenced the ‘*Ca*. L. solanacearum’ population size, based on the nearly equal ‘*Ca*. L. solanacearum’ genome copy number (based on Cq value) detected in psyllids reared at 18°C and 30°C. Based on Cq values, psyllids reared at both 18° and 30°C harbored one order of magnitude fewer ‘*Ca*. L. solanacearum’ genome copies, compared to PoP reared at the more ‘optimal temperature’ of 25°C (**Figure 1**).

### 3.2 Altered gene expression in response to temperature in ‘*Ca*. L. solanacearum’-infected and –uninfected potato psyllid

**Figure 2:**
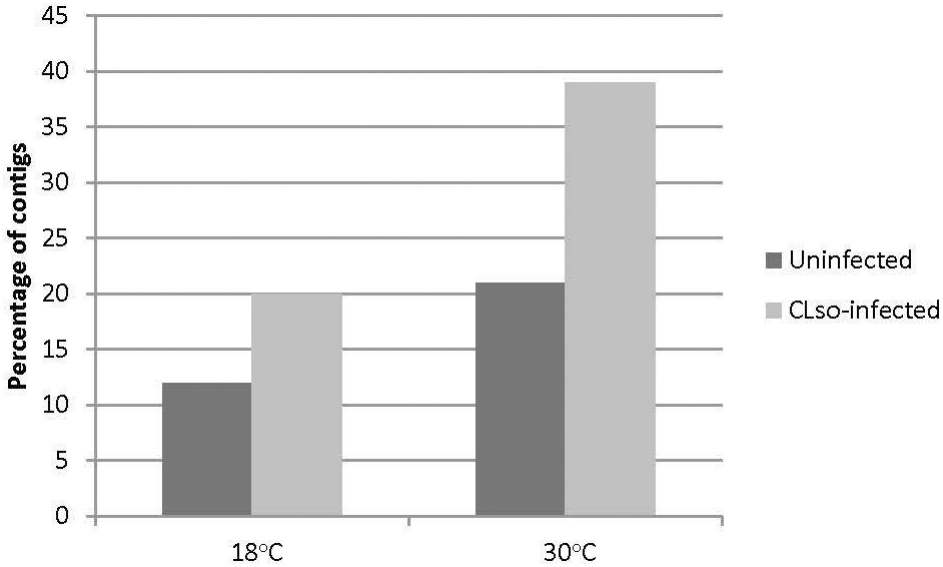
Percentage of transcripts with 2-fold or greater change in gene expression at 18°C or 30°C, to the moderate, herein considered ‘optimal’ temperature of 24°C, and 18°C to 30°C comparisons. The results indicate that more transcripts were differentially expressed at 30°C, but not at 18°C, when compared to 24°C, for the uninfected psyllids (black bars), and the ‘*Ca.* L. solanacearum’-infected psyllids (gray bars). The direct comparisons of 18°C to 30°C showed a trend similar to that observed for the 30°C and 24°C temperatures.

**Figure 3:**
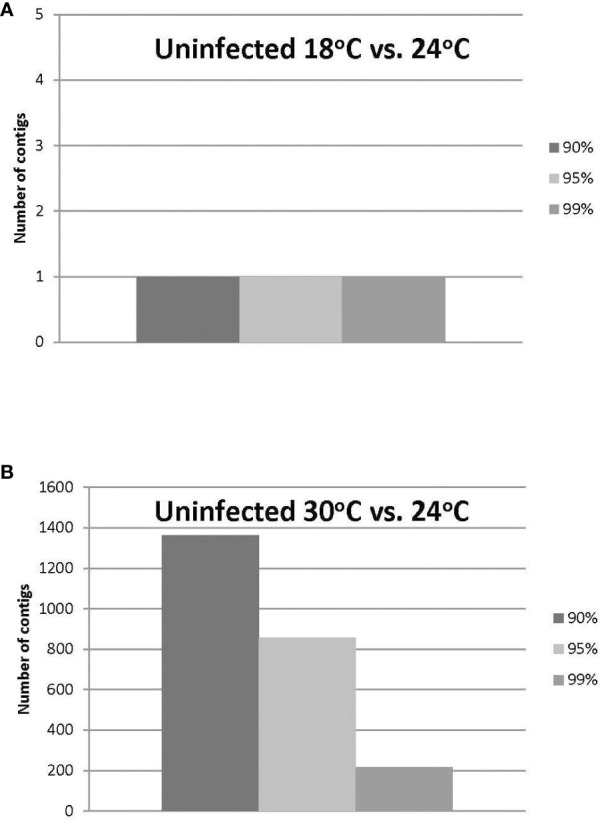
**(A, B)**. Number of uninfected potato psyllid transcripts with significant changes in gene expression at 18°C or 30°C, compared to the optimal temperature of 24°C at the different Boneferroni confidence intervals. More transcripts were affected by the highest **(B)** compared to the lowest temperature **(A)**, and fewer transcripts exhibited significant differential expression, with increasing confidence **(A, B)**.

The HiSeq 200 sequencing of psyllid RNA yielded approximately 1070 million pair-end high quality reads of 50 bp in length (**Table 1**), which were analyzed using the Q-Seq program in ArrayStar v14.0 (DNASTAR, Madison, WI), with default settings. The 25,634 potato psyllid transcripts (100%) were mapped. Among them, 3,142 transcripts (12%) exhibited a 2-fold change (r^2^ = 0.9591) or greater in gene expression at 18°C, compared to 24°C for the ‘*Ca.* L. solanacearum’-free (uninfected) psyllids (**Figure 2**). In particular, expression of transcript BcAN_04148; 60S ribosomal) was significantly differentially expressed (**Figure 3**; **Supplementary Table 1**). Comparisons of *B. cockerelli* gene expression profiles at 30°C and 24°C, indicated that 5,547 transcripts (21%) exhibited at least a 2-fold change (r^2^ = 0.8833) for ‘*Ca.* L. solanacearum’-free (uninfected) psyllids (**Figure 2**). Though the overall number of significantly expressed genes was reduced after the Bonferroni statistical analysis (**Figure 3**), a robust number of genes, or 858, showed at least a 2-fold change in gene expression due to temperature-related affects (95% confidence level) (**Supplementary Table 2**).

**Table 1:** Summary of transcript reads for potato psyllid libraries.

	Uninfected Potato Psyllid			Infected Potato Psyllid		
	18°C	24°C	30°C	18°C	24°C	30°C
Read Type	Single	Single	Single	Single	Single	Single
Read Length (bp)	50	50	50	50	50	50
Total Number of Reads	188,083,380	191,152,116	172,856,023	203,918,822	159,519,431	154,461,053

**Figure 4:**
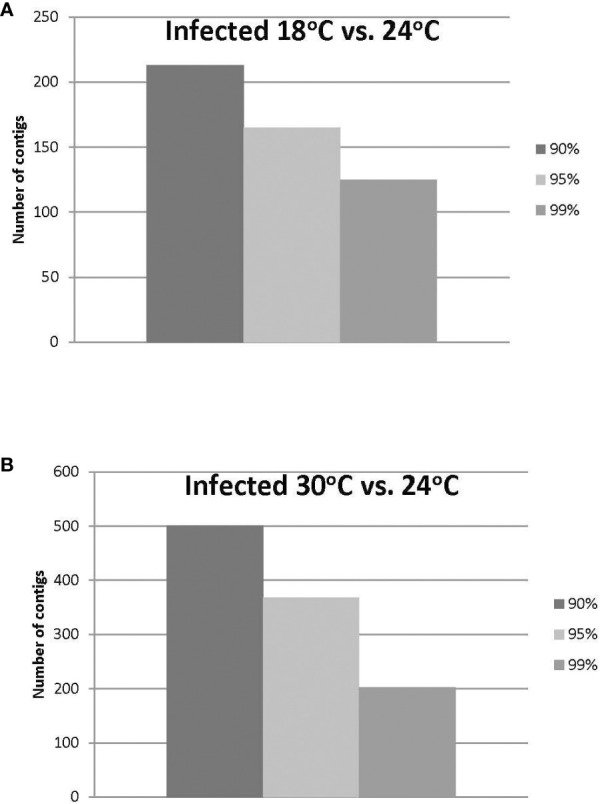
**(A, B)**. Number of ‘*Ca*. L. solanacearum’-infected potato psyllid transcripts with significant changes in gene expression at 18°C or 30°C, compared to the optimal temperature of 24°C at the different Boneferroni confidence intervals. More transcripts were affected by high temperature **(B)** than lower temperature **(A)**, and fewer transcripts exhibited significant differential expression, with increasing confidence **(A, B)**.

**Figure 5:**
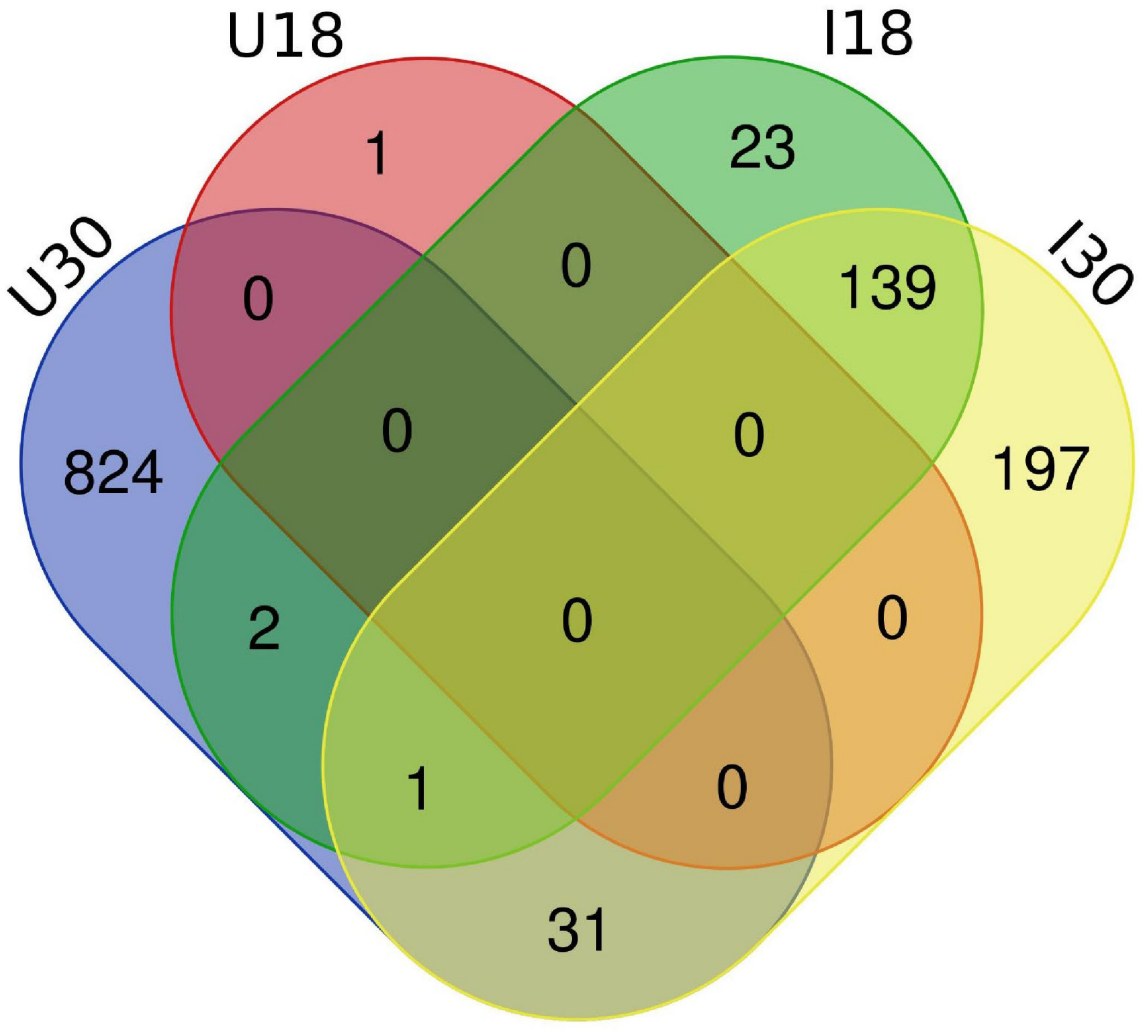
Venn diagram depicting the unique and overlapping genes between ‘*Ca.* L. solanacearum’ -infected and uninfected potato psyllids which had significant expressional differences (2-fold; 95% Bonferroni confidence interval). ‘*Ca*. L. solanacearum’-free psyllids had more differentially expressed transcripts at 30°C than ‘*Ca*. L. solanacearum’-infected psyllids. ‘*Ca*. L. solanacearum’-infected psyllids had more differentially expressed transcripts at 18°C than uninfected potato psyllids.

Approximately 20% of the transcripts expressed in the ‘*Ca*. L. solanacearum’-infected psyllids showed at least 2-fold change in expression at 18°C compared to 24°C (r^2^ = 0.8778), and nearly 40% at 30°C compared to 24°C (r^2^ = 0.8382) temperature comparisons (**Figure 2**). Of these, 165 and 368 transcripts were significantly differentially expressed (95% confidence; Bonferroni) at 18°C or 30°C treatments, compared to the 24°C temperature, respectively (**Figure 4**; **Supplementary Tables 3**, **4**). Further, approximately 87% (1,218) of the 1,392 differentially expressed genes exhibiting altered expression following low and high temperature treatments, respectively (**Supplementary Tables 1**-**4**), however, expression of certain genes was often found to apply exclusively to a specific temperature treatment over others (**Figure 5**). Regardless of the ‘*Ca*. L. solanacearum’-infection status, fewer genes were differentially expressed at the lowest temperature (18°C). However, based on the differential expression of 23 genes compared to one unique gene, respectively, the ‘*Ca*. L. solanacearum’-infected psyllids appeared to exhibit a greater sensitivity to heat stress than their *Ca*. L. solanacearum-free (uninfected) counterparts, (**Figure 5**). The 23 uniquely and significantly differentially expressed genes of ‘*Ca*. L. solanacearum’-infected psyllids reared at 18°C were identified (annotated) as mostly ribosome synthesis-related genes to increase protein synthesis. Another notable gene in this group was Acetyl-CoA (BcAN_14104), which is known to be involved in lipogenesis. The differential expression of this gene was associated with high and low temperature regimes and is consistent with the increased need for cuticular hydrocarbons during stress (26, 71). This observation is supported by a previous study in which ‘*Ca*. L. asiaticus’-infected *D. citri* exhibited mitochondrial dysfunction associated with changes in gene expression of *D. citri* genes involved in the citric acid cycle (TCA) (72). Strikingly, quite the opposite outcome was documented for *D. citri* adults exposed to the high temperature regime (30°C) when compared to 24°C treatment. Finally, based on the significantly greater number of differentially expressed and unique genes, e.g., 824 and 197, respectively, the expression profiles indicated that the ‘*Ca*. L. solanacearum’-free psyllids were much more sensitive to thermal stress than their ‘*Ca*. L. solanacearum’-infected counterparts (**Figure 5**).

### 3.3 Kyoto Encyclopedia of Genes and Genomes (KEGG) analyses of temperature-sensitive potato psyllid transcripts

**Figure 6:**
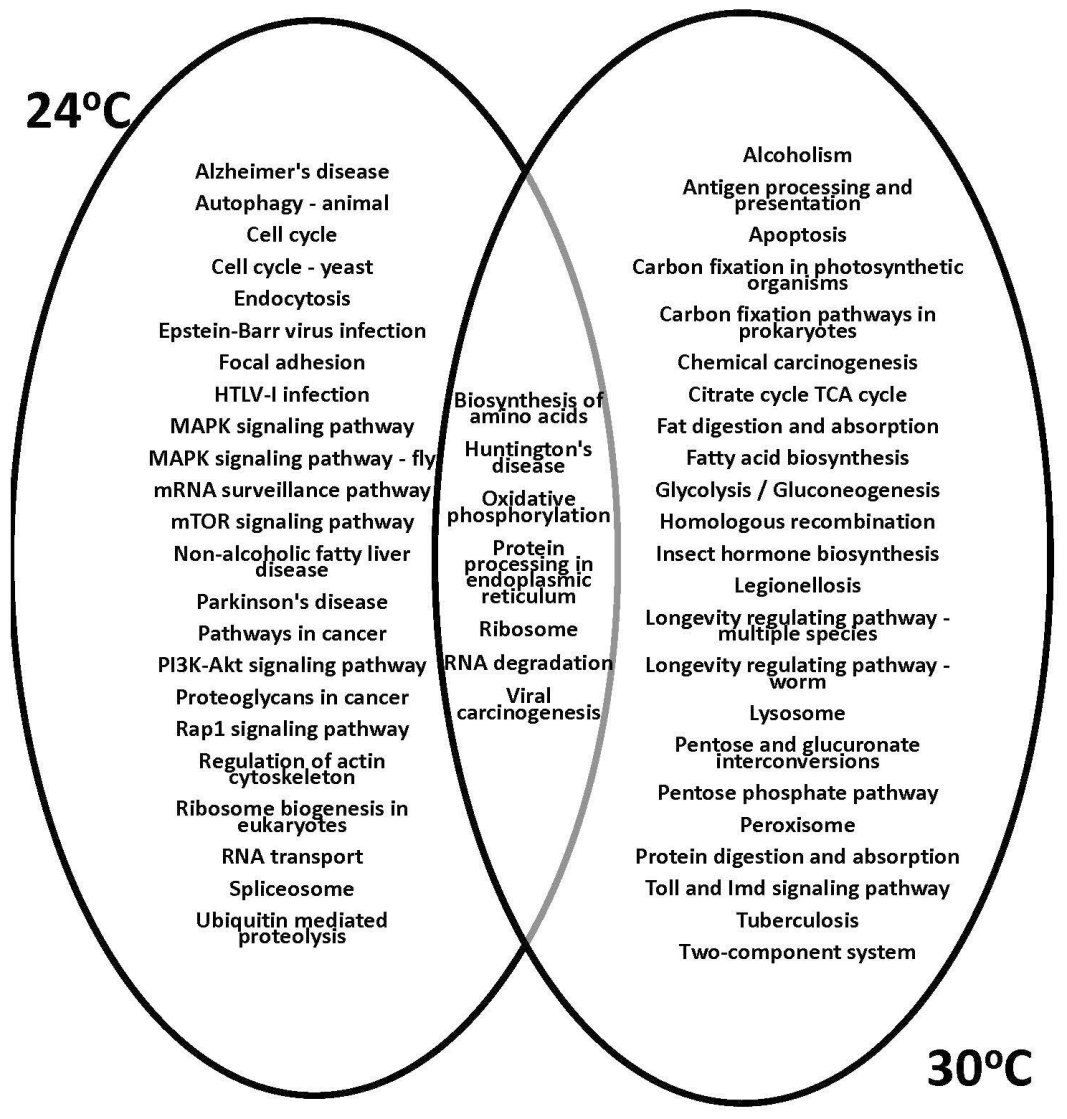
Kyoto Encyclopedia of Genes and Genomes (KEGG) analysis of genes significantly differentially expressed (2-fold; 95% Bonferroni confidence interval) at the 30°C temperature regime for the ‘*Ca*. L. solanacearum’-infected and uninfected potato psyllids. Venn diagrams show the shared and unique assigned pathways (among the top 30 non-metabolic KEGG pathways) between psyllids reared at 24°C and 30°C.

The KEGG analysis and gene function prediction revealed the context of specific pathways involved in physiological responses to temperature treatments. The 1,218 unique genes that showed altered expression of at least 2-fold (95% CI; Bonnferroni) in response to both low and high temperature treatments (**Figure 5**) were subjected to further analysis. The gene expression profiles showed that the greatest effect occurred at the highest temperature examined, 30°C, based on total number of differentially expressed psyllid genes. Subsequently, the KEGG functional assignments were compared for the ‘*Ca*. L. solanacearum’-infected and -uninfected adult psyllids reared at the putative optimal (24°C) and high (30°C) temperature treatments (**Figure 6**). Results showed that functions previously observed to be associated with pathogen invasion (65, 73–75) were over-expressed, particularly those with predicted involvement in endocytosis, Rap 1 signaling, and regulation of the actin cytoskeleton. Notably, these latter predicted functions were not observed among the transcripts of psyllids reared at 30°C.

## 4 Discussion

Host-pathogen dynamics are complex and not well-studied for the *B. cockerelli*-*’Ca.* L. solanacearum*’* pathosystem. However, environmental factors such as temperature and light are well-known to influence insect vector behavior. Recently, size and morphometric variation in psyllids associated with temperature change have been investigated (76). The effects of temperature on *B. cockerelli*-mediated *‘Ca.* L. solanacearum*’* transmission efficiency are not well-studied, nor are the cellular and tissue processes that underlie *‘Ca.* L. solanacearum*’* pathogenesis. Also of interest is how changes in *B. cockerelli* gene expression is manifest in extreme seasonal weather that may modulate *‘Ca.* L. solanacearum*’* multiplication/accumulation in the *B. cockerelli* host relative to vector competency. Further, understanding the relationship between *‘Ca.* L. solanacearum*’* load (accumulation), transmission efficiency, disease incidence, and disease severity could inform disease risk and knowledge-driven disease management.

The results for ‘*Ca.* L. solanacearum’ accumulation for *B. cockerelli* from qPCR analysis (**Figure 1**) were consistent with findings showing the significantly higher ‘*Ca*. L. asiaticus’ genome copy number in *D. citri* adults reared on citrus plants maintained at 25°C, compared to those reared on citrus maintained at 15°C or 35°C (32). However, for *B. cockerelli* the ‘*Ca.* L. solanacearum’ genome copy number at 15°C was significantly higher than for psyllids reared at 35°C. These results further support the differences seen in transmission efficiency, reported previously (5), were more likely due to increased, decreased, or neutral changes in psyllid gene expression as the result of exposure to changes in temperature. In a previous study, it was suggested that differences in transmission efficiency in the vector might be associated with differences in accumulation of ‘*Ca.* L. solanacearum’ in the individual psyllids and/or populations, however, it is expected that ‘*Ca.* L. solanacearum’ accumulation in psyllids would remain relatively constant within the *B. cockerelli* population collected from the same colony until they were exposed to differential temperature regimes, respectively. A greater understanding of differential gene expression in *B. cockerelli* experiencing temperature stress with respect to the dynamics associated with psyllid metabolism, reproduction, and transmission efficiency is needed. These parameters are the most influential in pest and disease risk models, particularly for potato psyllid, which are integral to determining the extent of ‘*Ca.* L. solanacearum’ spread that will occur in crops and overwinter in wild host plants.

A combination of biotic and abiotic stress may have shaped psyllid susceptibility or tolerance to *‘Ca.* L. solanacearum*’* infection, and perhaps benefitted, to manifest the initial invasion of solanaceous crops and wild host species by *‘Ca.* L. solanacearum*’*-infected *B. cockerelli*, which was observed for the first time in the mid-1980’s (14). Previously, the potato psyllid has been known to migrate annually from possibly as far south as Central America and southern Mexico, and to disperse north and northwesterly in the continental U.S. and Canada. Since the mid-1900’s the potato psyllid was reported colonizing potato crops and to cause a disorder known as psyllid yellows, which resulted in the development of symptoms that are distinct from that presently associated with ‘*Ca.* L. solanacearum*’* infection of potato. Because host plants recovered from symptoms when psyllid numbers were diminished on plants following insecticide application, a psyllid-transmissible pathogen could be ruled out as the causal agent of the disorder, which led to the hypothesis that psyllid yellows was caused by feeding damage, possibly associated with a salivary toxin. Thus *‘Ca.* L. solanacearum*’* is a relatively new pathogen that has been only recently associated with migrating *B. cockerelli* adults. This putative new ‘*Ca*. L. solanacearum’-psyllid pathosystem could well have emerged as a consequence of positive selection. This hypothesis is consistent with pathosystem that was first reported in ~2000 to the present, or at about the time estimated for the earliest temperature shifts that were experienced at equatorial/tropical and subtropical latitudes with the onset of climate change (3, 4, 14). In other scenarios, *‘Ca.* Liberibacter’ spp. have been associated with the trade-offs of increased fecundity with minimal effects on longevity (77).

Only minimal statistically significant changes in gene expression were observed for *‘Ca.* L. solanacearum*’*-free *B. cockerelli* reared at the sub-optimal temperature of 18°C, compared to expression profiles at 24°C, the proxy used herein for the predicted optimal temperature range of 22-26°C. Thus, psyllids experienced little to no discernable biological effects evident based on gene expression, at the low to mid-temperature ranges studied here. In contrast, at 30°C, *‘Ca.* L. solanacearum*’*-free adults exhibited statistically significant changes in gene expression, particularly among genes involved in abiotic and biotic stress responses. Interestingly, increased gene expression by *‘Ca.* L. solanacearum*’*-free *B. cockerelli* was greater than *‘Ca.* L. solanacearum*’*-infected psyllids for which twice the number of genes had altered expression profiles. Consequently, gene expression modulation associated with the physiological responsiveness of *B. cockerelli* is affected more by high temperature-induced stress than by lower than optimal temperatures. These observations suggest that *‘Ca.* L. solanacearum*’* infection and spread in *B. cockerelli* populations occurred and/or can shift from once potentially rare levels to apparently near fixation. In turn, the establishment of *‘Ca.* L. solanacearum*’* (A and B haplotypes) has provided an adaptive mechanism for combatting thermal stress, resulting in increased survival of *B. cockerelli* and widespread distribution of *‘Ca.* L. solanacearum*’* haplotypes among *B. cockerelli* populations since the emergence of zebra chip disease in Mexico and the western and central U.S. beginning in the late1990’s-2000 (5).

The predicted functions of the differentially expressed genes reported in this study are consistent with previous reports that have implicated abiotic stress genes, as well as aquaporin, which showed 2.8-fold increased expression in *‘Ca.* L. solanacearum*’*-infected psyllids reared at 30°C, compared to 24°C (48). In addition, several cathepsin-like genes (5- to 18-fold) and a phenol oxidase gene (3-fold) showed greater expression in *‘Ca.* L. solanacearum*’*-infected psyllids reared at 30°C compared to the simulated optimal temperature of 24°C. This is consistent with previous reports in which innate immune responses have been attributed to *‘Ca.* L. solanacearum*’*-*B. cockerelli* interactions associated with the circulative, propagative mode of transmission (65, 73, 78). Also, a cadherin-like gene, with predicted involvement in cell-to-cell interactions known in other pathosystems involving pathogen infections with host gut surfaces (79, 80) showed 3-fold increased expression in *‘Ca. L. solanacearum’*-infected psyllids reared at 30°C, compared 24°C. These observations suggest that the *B. cockerelli* innate immune system is more responsive to temperature stress in *‘Ca. L. solanacearum’* infected psyllids, to afford greater protection to *‘Ca.* L. solanacearum*’*-infected over *‘Ca.* L. solanacearum*’*-uninfected psyllids, which could result in a higher percentage of *‘Ca. L. solanacearum’*-infected potato psyllid in each population, under thermal stress. It also suggests that *‘Ca.* L. solanacearum*’* infection provides a protective effect to ensure survival of itself and the psyllid host and vector. Consistent with the differential transcript profiles, the predicted KEGG pathway gene functions have also been implicated in circulative, propagative, transmission of *‘Ca. L. solanacearum’*, namely, proteins involved in ribosome synthesis, endocytosis, and actin cytoskeleton regulation (81, 82). Specifically, the reduction or absence of expression of these transcripts in the potato psyllid vector maintained at 30°C, and the significantly lower transmission efficiency reported in previously published experiments conducted at 30°C (5), are taken together as evidence that Rap1 signaling, actin cytoskeleton remodeling, and endocytosis are critical for host-’*Ca*. L. solanacearum’ interactions, particularly during the early to mid-instar stage infection processes, that corresponds to the timeframe prior to profuse systemic infection and ‘acquisition’ or entry of viable ‘*Ca*. L. solanacearum’ into the potato psyllid salivary glands. These results point to these and other pathway-associated genes as potentially lucrative targets for therapies aimed at disrupting optimal ‘*Ca*. L. solanacearum’ gut invasion and systemic infection of the psyllid host, and result in lowered rates of pathogen transmission in the psyllid population as well as reducing tree-to-tree ‘*Ca*. L. solanacearum’ spread. Notably, KEGG pathway predictions for potato psyllids reared at the most extreme temperature, 30°C support results of previously published studies that reported increased expression of genes in the Lysosome and Toll and Imd signaling pathway (53), a pathway responsible for modulating early responses that might be anticipated in an insect host/vector to counter invasion and systemic infection of ‘*Ca*. L. solanacearum’.

These proteins are hypothesized to be important mediators of the initial gut invasion stages of the *B. cockerelli* host by *‘Ca.* L. solanacearum*’* (65, 83). Indeed, *‘Ca.* L. solanacearum*’* invasion of *B. cockerelli* exploits host endo-exocytic processes and cytoskeletal remodeling (84) as well as other cellular processes negatively affected by high temperatures that could ultimately result in a lowered accumulation of Liberibacter in the psyllid host which could feasibly be detrimental to *‘Ca.* L. solanacearum*’* survivability in the long term. Thus, blocking or disruption of these processes by RNAi for example, may result in reduced *‘Ca.* L. solanacearum*’* transmission frequency, while also lessening the protective effects to thermal stress afforded by *‘Ca.* L. solanacearum*’* to the psyllid host.

Finally, the abundance of differentially expressed genes, at 824 compared to 197, and the over-expression of genes encoding stress-responsive proteins in *‘Ca.* L. solanacearum*’*-infected compared to *‘Ca.* L. solanacearum*’*-free *B. cockerelli* indicated *‘Ca.* L. solanacearum*’* infection affords protection against thermal stress, effectively ensuring survival of the pathosystem and *‘Ca.* L. solanacearum*’* transmission to the plant host. A recent study *‘Ca.* L. solanacearum*’* of the A and B haplotypes of *B. cockerelli* showed that infection by each haplotype resulted in the differential expression of a unique suite of gut genes predicted to be involved in innate immunity. Further, the transcriptional responses in *‘Ca.* L. solanacearum*’* B infected *B. cockerelli* guts exhibited a more robust response than in psyllid guts infected with *‘Ca.* L. solanacearum*’* A (85).

The recent emergence of *‘Ca.* L. solanacearum*’*-*B. cockerelli* pathosystems approximately 20 years ago appears to have coincided with early effects of global warming in subtropical and mild temperate climes. This observation suggests that *‘Ca.* L. solanacearum*’*-infected *B. cockerelli* populations emerged in parallel with increasingly higher temperatures occurring in Central America ~1000 miles from the equator, where the earliest *B. cockerelli* outbreaks were observed (3, 86). Recent studies have shown that *‘Ca.* L. solanacearum*’*-infected psyllids are endemic in potato-growing areas of Mexico and Central America from where they migrate annually to the southwest/northwest and central parts of the U.S. and Canada. Until recent outbreaks of zebra chip and tomato vein-greening diseases emerged, *B. cockerelli* was thought to overwinter and migrate into the western U.S. and Canada south of the Rio Grande Valley, Texas (14). Apparently, the *‘Ca.* L. solanacearum*’*-free *B. cockerelli* populations previously associated with psyllids yellows disorder have only recently been replaced by the *‘Ca.* L. solanacearum*’*- *B. cockerelli* pathosystem, predominating in the western U.S. and Canada since the late 1990s (4, 14).

## 5 Conclusions

This study documents for the first time the effects of thermal stress on the gene expression of *‘Ca.* L. solanacearum*’*-infected compared to *‘Ca.* L. solanacearum*’*-free (uninfected) potato psyllids from a functional genomic perspective. Less than optimal temperatures negatively influenced *‘Ca.* L. solanacearum*’* invasion, multiplication (genome copy number), and potentially exocytosis from the gut to the blood, ultimately reducing the circulating *‘Ca.* L. solanacearum*’* cells available for entry into the salivary glands and/or oral cavity (40, 87, 88) from where the bacteria are transmitted to the psyllid host. The putative high temperature-associated reduced in *‘Ca.* L. solanacearum*’* accumulation in the psyllid vector is consistent with previously reported *in planta* observations. Indeed, symptom development was reported to be delayed in *‘Ca.* L. solanacearum*’*-infected plants maintained at low temperatures, while symptom development was accelerated in *‘Ca.* L. solanacearum*’*-infected plants maintained at higher than optimal temperatures. *In silico* expression and functional analyses pointed to several *B. cockerelli* genes that function naturally in cytoskeletal remodeling and are likely utilized by *‘Ca.* L. solanacearum*’* for invasion, and in psyllid immunity and other defenses. Expression of some of the proteins identified here during rearing of psyllids under prolonged conditions of thermal stress, can be modulated by *‘Ca.* L. solanacearum*’*-infection (65, 73, 89, 90) to enhance *‘Ca.* L. solanacearum*’* invasion of and pathogenesis in young psyllid instars, and *‘Ca.* L. solanacearum*’* multiplication and systemic infection of *B. cockerelli*. Other *B. cockerelli* gene products are implicated as *‘Ca.* L. solanacearum*’*-effector interactors with predicted functions in attachment/invasion/exocytosis, and potentially salivary glands entry (65, 73, 84, 91, 92). The RNAseq differential expression profiles reported here provide the first snapshots into host-pathogen thermal adaptation mechanisms and highlight key biochemical pathways in its insect host, apparently essential for *‘Ca.* L. solanacearum*’* survival outside of the plant, and for psyllid-to-plant transmission. Whether naturally occurring extreme temperatures would be expected to result in a shift or curtailment of extant *B. cockerelli*-*’Ca.* L. solanacearum*’* habitats remain to be seen. The results of this study are expected to provide useful insights into risk assessment and strategies for psyllid vector-*’Ca.* L. solanacearum*’* control pertaining to the potential for the different *B. cockerelli*/*’Ca.* L. solanacearum*’* haplotype combinations to adapt to changing temperature and weather/climatic conditions.

## Data availability statement

The raw data reads used in this study are deposited in the NCBI SRA repository under Bioproject PRJNA252003.

## Author contributions

TF: Formal analysis, Investigation, Validation, Writing – original draft, Writing – review & editing. JM: Conceptualization, Funding acquisition, Project administration, Supervision, Writing – review & editing. JB: Conceptualization, Project administration, Supervision, Visualization, Writing – review & editing.

## Supplementary material

Supplementary Table 1 List of differentially expressed contigs between ‘*Ca*. L. solanacearum’-uninfected psyllids reared at 18° and 24°C.

Supplementary Table 2 ‘*Ca*. L. solanacearum’-uninfected psyllids reared at 30° and 24°C.

Supplementary Table 3 ‘*Ca*. L. solanacearum’-infected psyllids reared at 18° and 24°C.

Supplementary Table 4 ‘*Ca*. L. solanacearum’-infected psyllids reared at 30° and 24°C.
